# How Affective Science Can Inform Behavioral Public Policy

**DOI:** 10.1007/s42761-024-00264-y

**Published:** 2024-08-26

**Authors:** Daniel Västfjäll, Erkin Asutay, Gustav Tinghög

**Affiliations:** 1 Department of Behavioural Sciences and Learning, Division of Psychology, Jedi-Lab, Linköping University, 581 83 Linköping, Sweden; 2 Decision Research, Eugene, USA; 3 Department of Management and Engineering, Division of Economics, Jedi-Lab, Linköping University, 581 83 Linköping, Sweden

**Keywords:** Affective paternalism, Affect, Behavioral public policy

## Abstract

In this commentary, we expand on the special issue themes of applied affective science, ecologically valid data and application, and the need for transdisciplinary collaboration by discussing and exemplifying how affective science can inform behavioral public policy.

Affective science has a great potential to inform policies and governmental decision-making to help achieve behavior change, better people’s lives, and mitigate societal and environmental disasters caused by human behavior. As argued by Shiota et al. (2023) in the introduction to the special issue of the future of affective science, affective science needs to reconnect basic and applied affective science. Ferrer & Gillman (2023) point out that applying affective science approaches to behavior in various real-life contexts may enable us to generate new theories, methods, and data (Wilson-Mendenhall & Holmes, 2023) to test them. Affective sciences “in the wild” is also a needed step to get ecologically valid data on how affect is constructed, what emotions people experience in their everyday lives, and how it impacts real-life behavior.

We agree with Ferrer and Gillman (2023) that theories of behavior change are mostly affectless and often not very detailed or precise in the operationalization or role of affect. To make affective science more relevant to behavioral public policy (and vice versa), we believe that (1) clear, testable, and theories of how affect influence behavior; (2) defined areas where such theories can be applied in an ethical way (Ferrer and Gillman, 2023); and (3) integration with other disciplines (Shiota et al., 2023) are needed. Here we present such an example building on research in affective science, judgment, and decision-making/behavioral economics, and public policy: *Affective Paternalism.*

## Affective Paternalism

Behavioral economics have had a massive impact on public policy. Theories of heuristics and biases (Kahneman et al., 1982) and System 1 and 2 dual-process theories (Kahneman, 2011) has been the theoretical foundation for “nudging.” Nudges typically involve changes by “choice architects” to the “choice architecture” (Thaler & Sunstein, 2008) of a decision. Choice architecture can best be described as the design of how options are presented to decision-makers and its subsequent impact on decision-making. Thus, choice architecture can be anything that makes an option weighted differently in decision or change the likelihood to be chosen/not chosen. An important assumption of choice architecture is that preferences are not stable values but constructed based on information available at the time of the decision (Slovic & Västfjäll, 2010). Typical tools for choice architects are changing the default option of a choice, presenting options differently, and incentivizing choices (Thaler et al., 2012). Ideal nudges benefit both the decision-maker and others, and many people accept nudge interventions if they feel that they are not infringing on free choice (Hagman et al., 2015). Nudges should encourage pro-environmental and prosocial behavior “…without forbidding any options or significantly changing their economic incentives*…*.” (Thaler & Sunstein, 2008, p. 6), and can for example be achieved by providing descriptive social norms (e.g., how many other donate to charitable cause; Andersson et al., 2021). While such behavioral interventions have proven somewhat effective in temporarily improving or changing certain behaviors, the perhaps most limiting factor for advancement of both theory and application of nudges is that the psychological foundations on which they rest often ill specified and poorly understood. It may even be argued that the common tools for choice architecture are largely based on outcomes and not mechanisms. While the theories underpinning the nudge framework is vaguely related to affect (e.g., system 1 intuitions: Kahneman, 2011), a nuanced use of precise affective science theories is still lacking. But how can theories of affect be used to inform public policy beyond what is done in the nudge framework?

Let’s consider a domain where the affective drivers of behavior change can be applied relatively easily: The case of impactful behaviors and scope/impact neglect. People are often insensitive to scope or magnitude in their responses to and evaluations of many of the largest societal challenges. In the domain of life saving many people agree that, if every statistical life is of equal value, the value of saving N lives should be N times the value of saving one life (Slovic & Västfjäll, 2010). Yet, people respond very differently to this principle. For example, the picture of Aylan Kurdi—one identified Syrian child—whose body had washed up on the Turkish shore on 2 September 2015 became an iconic, evocative image of the Syrian refugee crisis. Aylan’s image moved people around the world to act and prompted international responses to the Syrian crisis far more than the tragedy of hundreds of thousands of deaths and displaced Syrians whose plight was published by the media around the world for months (Slovic et al., 2017). Most people would agree that hundreds of thousands of deaths (including many thousands of children) is greater than the tragedy of one dead child and should motivate donations and international response. But the actual responses of people and their governments fell far short of what would be expected from this normative perspective—a form of scope neglect (Slovic et al., 2017; Västfjäll & Slovic, 2020). People do not base their decision on whom to help on how help can be most effectively provided (i.e., help the most) and this tendency appears to be driven by affect (good-bad feelings such as warm glow experienced when doing a “good thing” or specific emotions like fear, compassion, guilt, anger, anxiety, hope, etc., Västfjäll & Slovic, 2020). Importantly, both positive and negative affect/emotions may either motivate or demotivate behavior depending on the context (Ferrer & Gillman, 2023), but is still often relatively insensitive to objective scope or impact (Västfjäll & Slovic, 2013).

We therefore argue that a potentially more successful way to increase positive behaviors for self (health) and other (prosocial giving and pro-environmental behavior) may be to apply affective science findings to help people *refocus people’s efforts from low impact ones to high impact ones* (Asutay et al., 2023; Nielsen et al., 2021). In the domain of humanitarian aid, there have been efforts to increase altruism through cognitive mechanisms so that people redirect their efforts to the most cost-effective cause (Singer, 2009). We propose that affect, in a similar way, can be redirected to encourage pro-self, prosocial, and pro-environmental actions. Interventions designed to either weaken affect (e.g., through affect inductions, imagery, narratives rather than numbers in communicating risks/benefits; using the affective component of social emotion norms; Vishkin & Tamir, 2023) for low-impact actions and increase affect for high impact actions (or induce affect for affect-neutral options; which may be qualitatively different than just the mere absence of positive and negative affect; Gasper, 2023) can be designed based on the vast knowledge on emotion causation and affect induction generated in the affective sciences in the past 50 years. Using nudge terminology (Thaler & Sunstein, 2008), we can complement choice architecture (which typically capitalize on cognitive heuristics and biases such as availability, representativeness, and anchoring) with “affective architecture” to design environments that are conducive to elicit affect and emotions that motivate action (rather than changing the decision directly). A benefit of this approach over traditional affectless choice architecture is that positive (warm glow) and negative (cold prickle) affect associated with actions may, in addition to affecting a decision one time, have sustained effects on decisions through reinforcement learning and positive feedback loops (Asutay et al., 2024; Brosch, 2021; Schneider & van der Linden, 2023). As decisions often are motivated by hedonic goals, applying and expanding emotion regulation theories (Petrova & Gross, 2023) to affect architecture may be a promising possibility.

Taken together, while the traditional choice architecture tools focus on restructuring the environment to capitalize on cognitive shortcuts, the tools of affect architecture are reliable ways to induce affect through information presentation, mechanistic frameworks for emotion causation and construction, and accounting for individual differences in affect experience and regulation. Interventions based on choice architecture have been criticized for being a “one-nudge-fits-all” approach (Hagman et al., 2015) and thus, an approach based on affective science and its focus on the idiosyncrasies of affective experience has great potential to increase the effectiveness of nudges.

Our approach may a first sight be at odds with nudging and the notion of *libertarian paternalism*—the idea that behavioral interventions should nudge people to make decisions that they intended to do or don’t mind doing (Thaler & Sunstein, 2008). *Affective Paternalism* instead helps people focus their affect toward actions of high impact—actions they are already undertaking but are doing so in an suboptimal way. So, while affective paternalism in one sense can be seen as infringing on freedom as affect is induced (note this is already done in many existing nudging interventions, e.g., images on tobacco packing), it does not force people to make decisions they do not want to make or force them to give up behaviors they like. Importantly, their overall affective well-being will remain unchanged (because of scope neglect in affective responses people feel equally good about their actions). That is, if positive associations for one low impact behavior is “refocused” to a high impact behavior (e.g., by interventions aimed at increasing the positive affect of the high impact, and reducing the affect of the low-impact behavior), affective paternalism would be largely consistent with the welfare aspect of nudges: the individual life net effect on well-being should aim to be zero (or positive) of such interventions, but have massive positive consequences for society.

The example given here is focused on situations where individuals are affectively indifferent between alternatives (scope/impact neglect) but is possible to apply to other context and situations where the dynamical nature of affect needs to be understood to harness its power for behavior change. We recently outlined a high-stakes scenario where affective paternalism and affect architecture could benefit both the decision-maker and the choice architect; shared patient-doctor decision making (Tinghög et al., 2024). Many patients experience strong affect and emotions when they learn about the mortality risks associated with various cancer treatments. But that affective response is often rather insensitive to the survival rate associated with each option (probability neglect; Slovic, 2007). Thus, acknowledging the patients’ affect and helping to induce differentiated affective responses to different treatment options (e.g., by utilizing knowledge on affect induction to visualizing different outcomes to increase sensitivity to probability through affect-richness; Västfjäll & Slovic, 2013) could be a tool where affect is used rather than avoided in shared decision-making. Patients, while knowing that the information is critical for their health, feel that the information is unpleasant and therefore avoid it. Helping the patient to confront negative affective information so that it not avoided and instead used in decisions (for example, through emotion regulation strategies such as reappraisal; Langley et al., 2023; Petrova & Gross, 2023) is central. Affective science methods provide the tools to do this, while traditional choice architecture tools such as incentives, defaults, and affectless restructuring of information may be less effective in achieving this goal.

In the examples given here, we believe that individual and societal benefits outweigh the risk for the individual, but it is easy to imagine situations where the ethics of affective interventions are less clear. Acceptance among those subjected to the interventions (Hagman et al., 2015) should also be taken into consideration. However, as people tend to be poor at forecasting their future emotions and understimate hedonic adaptation (Fredrick & Lowenstein, 1999), it is possible that people overestimate how positive or negative they will feel about a behavior change intervention before they experienced it. Respectfully considering individual autonomy and present and future preferences while weighing societal benefits of any given intervention is central to the success of affect architecture.

Taken together, we believe that affective science theories and methods can be applied in an ethical way to a much larger extent than what is done today in behavioral public policy. While our proposal is preliminary and incomplete, it is an example of how affective science can inform behavioral public policy—something that is needed to both forward the field of affective science and to enable more effective behavioral change interventions. We call for transdisciplinary integration of the fields of affective science, behavioral economics, and public policy (and other related fields) to take the step needed to achieve behavior change for both individual and societal good at scale. Research funding enabling transdisciplinary work and collaboration with neighboring fields (e.g., behavioral economics) is required step for affective science as a field to make this leap (Simmons et al., 2023). But a deeper discussion of the ethical implications of a marriage between affective science and behavioral public policy is also needed before this step is fully taken. We hope our proposal can stimulate further discussion of how affective science can be applied in different contexts to stimulate both theory development and use of the knowledge generated in our field for individual and societal good.

## Additional Information

### Data Availability

No datasets were generated or analysed during the current study.
